# Ensemble Transductive Propagation Network for Semi-Supervised Few-Shot Learning

**DOI:** 10.3390/e26020135

**Published:** 2024-01-31

**Authors:** Xueling Pan, Guohe Li, Yifeng Zheng

**Affiliations:** 1 Beijing Key Lab of Petroleum Data Mining, Department of Geophysics, China University of Petroleum, Beijing 102249, China; 2020310415@student.cup.edu.cn; 2 College of Information Science and Engineering, China University of Petroleum, Beijing 102249, China; 3 School of Computer Science, Minnan Normal University, Zhangzhou 363000, China; zhengyifengja@gmail.com; 4 Key Laboratory of Data Science and Intelligence Application, Fujian Province University, Zhangzhou 363000, China

**Keywords:** few-shot learning, meta learning, graph semi-supervision, label propagation, Gaussian kernel function, D-S evidence theory

## Abstract

Few-shot learning aims to solve the difficulty in obtaining training samples, leading to high variance, high bias, and over-fitting. Recently, graph-based transductive few-shot learning approaches supplement the deficiency of label information via unlabeled data to make a joint prediction, which has become a new research hotspot. Therefore, in this paper, we propose a novel ensemble semi-supervised few-shot learning strategy via transductive network and Dempster–Shafer (D-S) evidence fusion, named ensemble transductive propagation networks (ETPN). First, we present homogeneity and heterogeneity ensemble transductive propagation networks to better use the unlabeled data, which introduce a preset weight coefficient and provide the process of iterative inferences during transductive propagation learning. Then, we combine the information entropy to improve the D-S evidence fusion method, which improves the stability of multi-model results fusion from the pre-processing of the evidence source. Third, we combine the L2 norm to improve an ensemble pruning approach to select individual learners with higher accuracy to participate in the integration of the few-shot model results. Moreover, interference sets are introduced to semi-supervised training to improve the anti-disturbance ability of the mode. Eventually, experiments indicate that the proposed approaches outperform the state-of-the-art few-shot model. The best accuracy of ETPN increases by 0.3% and 0.28% in the 5-way 5-shot, and by 3.43% and 7.6% in the 5-way 1-shot on miniImagNet and tieredImageNet, respectively.

## 1. Introduction

Deep learning is widely used in many practical applications, such as speech recognition [1], computer vision [2], and semantic segmentation [3]. It is data-driven and relies on a large amount of labeled data to train a model. However, in some scenarios, labeled data are costly to obtain. Therefore, how to utilize the limited labeled data to construct a reliable model is very important. Nowadays, inspired by human learning and utilizing prior knowledge to learn new concepts via only a handful of examples, few-shot learning (FSL) has attracted much more attention.

Few-shot learning has been divided into three categories, including augmentation, metric learning, and meta-learning. FSL usually adopts an episodic training mode. Each episodic training includes a support and a query set. The support set is constructed by randomly selecting *K* categories and *N* samples in each selected category from training data, namely the *K*-way *N*-shot. The query set is also randomly sampled from the *K* categories, but it has no intersection with the support set.

For augmentation approaches, they aim to increase the number of training samples or features to enhance data diversity. However, some basic augmentation operations need to be improved in the process of model training, such as rotating, flipping, cropping, translating, and adding noise into images [4,5]. With the development of deep learning, more sophisticated algorithms customized for FSL were proposed. Dual TriNet mapped the multi-level image feature into a semantic space to enhance the semantic vectors using the semantic Gaussian or neighborhood approaches [6]. ABS-Net established a repository of attribute features by conducting attribute learning on the auxiliary dataset to synthesize pseudo feature representations automatically [7].

Metric learning is designed to learn a pairwise similarity metric by exploiting the similarity information between samples. It means a similar sample pair has a high similarity score and vice versa. Matching Nets performed full context embeddings by adding external memories to extract features. It measures the similarity between samples via the cosine distance [8]. Proto Net constructed a metric space by computing distances between the prototype representations and test examples [9]. AM3 incorporated extra semantic representations into Proto Net [10]. TSCE utilized the mutual information maximization and ranking-based embedding alignment mechanism to implement knowledge transfer across domains, which maintains the consistency between the semantic and shared spaces, respectively [11]. Moreover, TSVR made the source and target domains have the same label space to quantify domain discrepancy by predicting the similarity/dissimilarity labels for semantic-visual fusions [12]. K-tuplet Nets changed the NCA loss of Proto Net into a K-tuplet metric loss [13]. The drawback of the above algorithms is that they cannot learn enough transferable knowledge in a small number of samples to enhance the model’s performance.

Meta-learning approaches aim to utilize the transferring experience of the meta-learner to optimize a base learner. It is divided into three categories: Learn-to-Measure [14,15,16], Learn-to-Finetune [17,18], and Learn-to-Parameterize [19,20,21]. MAML learned a suitable initialization parameter via a multi-task training strategy to guarantee its generalization [22]. TAML utilized an entropy-maximization reduction to address the over-fitting problem [23]. DAE employed a graph neural network based on a denoising auto-encoder to generate model parameters [19]. However, the above solutions should further consider the related information between the support set and the query set. Even more importantly, learning a base learner for few-shot tasks is easy to overfit, which results in high-variance or low-confidence predictions by lacking training data [24].

Nowadays, some researchers focus on measuring the relations between the support and the query instances via transductive graph theory. The transductive graph-based approaches [25,26,27] can effectively obtain the labels of the query set based on a few labeled samples. The main idea is that, regarding the samples of the support set and the query set as graph nodes, the nearest neighbor relationship between the support set and the query set is utilized for joint prediction to supplement the lack of label information. TPN employed the Gaussian kernel function to calculate the similarity as the weight to build a k-nearest neighbor graph (KNN-Graph), which uses the label propagation algorithm to transductively propagate labels between the support and query examples [25]. The drawback is that it may divide all graph vertices into a vast community or trap them in a local maximum to affect the stability and robustness of a model.

To address the above problems, we propose an Ensemble Transductive Propagation Network (ETPN). Firstly, two types of ensemble strategies are proposed, based on homogeneous and heterogeneous algorithms. These are referred to as Ho-ETPN (Homogeneous Ensemble Transductive Propagation Network) and He-ETPN (Heterogeneous Ensemble Transductive Propagation Network), respectively. Transductive inference, based on a graph, is used to extract valuable information shared between support-query pairs for label prediction. This approach circumvents the intermediate problem of defining a prediction function on an entire space in inductive learning. Secondly, a novel fusion strategy is proposed, based on an improved D-S evidence theory, to enhance the robustness of our proposal. The improved D-S evidence fusion approach first uses the Bhattacharyya distance to construct a conflict matrix between the mass function, and then uses this conflict matrix to obtain the support matrix. It then combines information entropy to recalculate the mass weight, realizing the pre-processing of the evidence source. This enhances the robustness and stability of few-shot classification. Thirdly, we propose an improved ensemble pruning approach to select individual learners with higher accuracy to participate in the integration of the few-shot model results. It employs the L2 norm to make the model more stable to small changes in the input and improve the model’s robustness.

In summary, the key contributions of our approaches are summarized as follows:

1. *Ensemble framework*: Based on the individual graph learner framework, we propose two ensemble strategies including the homogeneous and heterogeneous models, named Ho-ETPN and He-ETPN, respectively. Moreover, during transductive propagation learning, we add the preset weight coefficient and give the process of iterative inferences.
2. *Ensemble pruning*: Proposing an improved ensemble pruning method to conduct the selective results fusion by screening the individual learner with higher accuracy.
3. *Combination strategy*: An improved D-S evidence aggregation method is proposed for comprehensive evaluation. To the best of our knowledge, it is the first work that explicitly considers the D-S evidence theory in few-shot learning.
4. *Effectiveness*: Extension experiences about supervised and semi-supervised conducted on miniImageNet and tieredImageNet datasets show that our solution yields competitive results on a few-shot classification. More challenging is that distracted classes are introduced during the process of the semi-supervised experiment.

## 2. Related Work

(1) Transductive Graph Few-shot Learning

Recently, few-shot learning has become one of the hot spots. Transductive inference employs the valuable information between support and query sets to achieve predictions [25]. In a data-scarce scenario, it has been proven to improve the performance of few-shot learning over inductive solutions [28,29,30]. TPRN treated the sample relation of each support–query pair as a graph node, then resorted to the known relations between support samples to estimate the relational adjacency among the different support–query pairs [31]. DSN proposed an extension of existing dynamic classifiers by using subspaces and introduced a discriminative formulation to encourage maximum discrimination between subspaces during training, which avoids over-performing and boosts robustness against perturbations [32]. Huang et al. proposed PTN to revise the Poisson model tailored for few-shot problems by incorporating the query feature calibration and the Poisson MBO (Merriman–Bence–Osher) model to tackle the cross-class bias problems due to the data distribution drift between the support and query data [26]. EGNN exploited the edge labels rather than the node labels on the graph to exploit both intra-cluster similarity and inter-cluster dissimilarity to evolute an explicit clustering [33]. Unlike the above methods, in this paper, we adopt the transductive graph approach to construct the ETPN model. It leverages the related prior knowledge between support and query sets during the test phase and a novel fusion strategy to address the issue of high variance and over-fitting.

(2) Semi-supervised Few-shot Learning

Moreover, it is difficult to annotate samples in many fields, such as medicine, military, finance, etc. Thus, semi-supervised few-shot learning (SSFSL) approaches are proposed to leverage the extra unlabeled data to enhance the performance of few-shot learning. LTTL proposed a self-training model, which utilizes cherry-picking to search for valuable samples from pseudo-labeled data via a soft-weighting network [34]. PRWN proposed prototypical random walk networks to promote prototypical magnetization of the learning representation [35]. BR-ProtoNet exploited unlabeled data and constructed complementary constraints to learn a generalizable metric [36]. In this paper, we adopt transductive inference to utilize unlabeled data and distractor classes irrelevant to the classification task to boost robustness against perturbations.

(3) Ensemble Few-Shot Learning

Ensemble learning is widely used in classifications to enhance the generalization ability and robustness of models. Therefore, many researchers have applied the ensemble framework to few-shot learning. The main idea is to adopt a combination approach to reduce the over-fitting problem and enhance the stability of the model. DIVERSITY investigated an ensemble approach for training multiple convolutional neural networks (CNNs). Each network predicts class probabilities, which are then integrated by a mean centroid classifier constructed for each network. Moreover, it introduced penalty terms allowing the networks to cooperate during training to guarantee the diversity of predictions [37]. EBDM divided the feature extraction network into shared and exclusive components. The shared component aims to share and reduce parameters in the lower layers, while the exclusive component is designed to be unique to each learner in the higher layers [38]. HGNN proposed a novel hybrid GNN of a prototype and instance to address overlapping classes and outlying samples, respectively [39]. E^3^BM introduced a Bayes model for each epoch, which leverages innovative hyperprior learners to learn task-specific hyperparameters and enhances model robustness [40]. However, the existing integration strategies mainly adopt a max-voting strategy without considering information uncertainty. Different from the above methods, we propose an improved D-S method to solve the above problem by preprocessing the data source; moreover, we improved the ensemble pruning method to perform a selective ensemble with better accuracy.

The contribution of our algorithm is summarized in Table 1, including transduction inference (trans_inference), semi-supervised few-shot learning (SSFSL), ensemble, ensemble pruning, and information uncertainty (infor_uncertainty).

## 3. Problem Definition

Given a label set $C=\left\{c_{j}|j=1,2,\ldots ,N\right\}$, $c_{j}$ represents the label (i.e., a discrete value). $S=\left\{s_{i}|i=1,2,\ldots ,n\right\}$ denotes a sample set, $s_{i}=\left(x_{i},y_{i}\right)$ represents a sample, $x_{i}$ is the attribute values set, $∥x_{i}∥$ denotes number of dimensions, namely, $x_{i}=\left(x_{i}^{1},x_{i}^{2},\ldots ,x^{∥x_{i}∥}\right)$. If $y_{i}\in C$, $s_{i}$ represents labeled samples, otherwise $s_{i}=\left(x_{i},\_\right)$ represents unlabeled samples. Sample sets are divided into supervised represented $S_{sup}$ and unsupervised sample sets represented by $S_{uns}$, thus, $S=S_{sup}\cup S_{uns}$, where $S_{sup}=\left\{\left(x_{i},y_{i}\right)|\left(x_{i},y_{i}\right)\in S,y_{i}\in C\right\}$, $S_{uns}=\left\{\left(x_{i},\_\right)|\left(x_{i},\_\right)\in S\right\}$.

For $S_{sup}$, let X⟶C denote the process of predicting the labels by the classifier *F* for training samples, where $X=\{x_{i}|\left(x_{i},y_{i}\right)\in S_{sup}\}$, namely $\forall \left(x_{i},y_{i}\right)\in S_{sup}$, $\tilde{y_{i}}=F\left(x_{i}\right)$. The accuracy rate of a classifier *F* is defined as $Acc\left(S_{sup},F\right)=\frac{\left|\{\left(x_{i},y_{i}\right)|\left(x_{i},y_{i}\right)\in S_{sup},\tilde{y_{i}}=F\left(x_{i}\right),\tilde{y_{i}}=y_{i}\}\right|}{|S_{sup}|}$. Supervised machine learning is the process of obtaining a classifier *F* from $S_{sup}$.

For *F* and $S_{uns}$, obtaining $S_{uns\to sup}$ from $S_{uns}$ is the process of adding annotations, which can be defined as $S_{uns\to sup}=\left\{\left(x_{i},\tilde{y_{i}}\right)|\left(x_{i},\_\right)\in S_{uns},\tilde{y_{i}}=F\left(x_{i}\right)\right\}$. For $S=S_{sup}\cup S_{uns}$ and X⟶C, where $X=\{x_{i}|\left(x_{i},y_{i}\right)\in S_{sup}or\left(x_{i},\tilde{y_{i}}\right)\in S_{uns\to sup}\}$, semi-supervised machine learning is the process of obtaining *F* from *S*.

For $S=S_{T}\cup S_{V}$, where $S_{T}$ is the train set, $S_{V}$ is validation set. *F* is learned from $S_{T}$. The validation is the process of calculating $Acc(S_{V},F)$.

Few-shot learning constructing models generally adopt episodic training mode. According to the above notations, the episodic training (K-way, N-shot) is defined as follows: let the label set of the support set denote $C_{K}\subseteq C$, $|C_{K}|=K$, $\forall C_{K},\exists y\in C_{K}$, $S^{N}\subseteq S$, $|S^{N}|=N$, $s\in S^{N}$, s=(x,y); the support set is defined as $T^{K\cdot N}=\left\{S_{i}^{N}|i=1,2,\ldots ,K\right\}$, $\exists y\in C_{K}$ and the query set is defined as $Q^{M}\subseteq S$, $|Q^{M}|=M$, $q\in Q^{M}$, $\forall S_{i}^{N}\in T^{K\cdot N}$, $q\notin S_{i}^{N}$.

For $S_{uns\to sup}=\left\{\left(x_{i},\tilde{y_{i}}\right)|\left(x_{i},\_\right)\in S_{uns},\tilde{y_{i}}=F\left(x_{i}\right)\right\}$, the interference sets consist of the distractor classes irrelevant to the target tasks [42,43]. It is added in the support and query sets to boost model robustness.

## 4. Methodology

**Figure 1:**
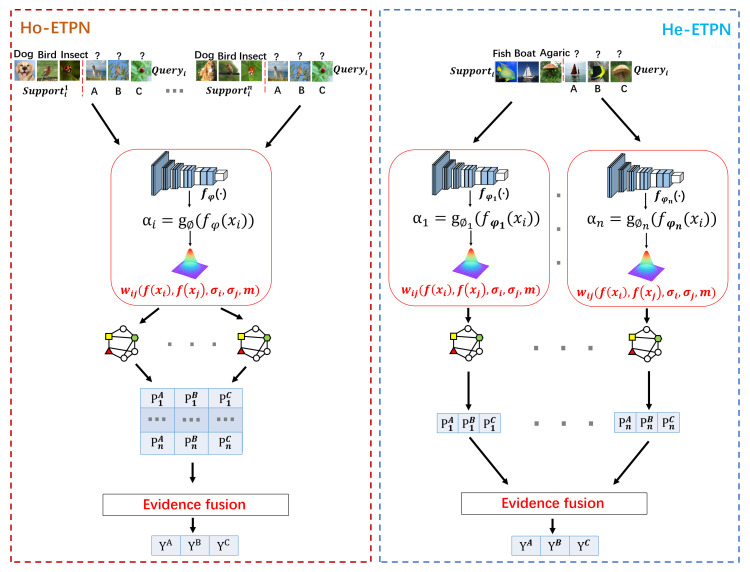
The overall framework diagram of the model.

In this paper, we propose the ensemble transductive propagation network (ETPN). The whole framework of the ensemble model is shown in Figure 1. For ETPN, we propose Ho-ETPN and He-ETPN models according to different ensemble approaches. Additionally, we incorporate a preset weight coefficient and compute iterative inferences during transductive propagation learning. Moreover, an improved D-S evidence fusion strategy is proposed for comprehensive evaluation. Meanwhile, we improve the ensemble pruning method to screen individual learners of higher accuracy to conduct fusing.

**Figure 2:**
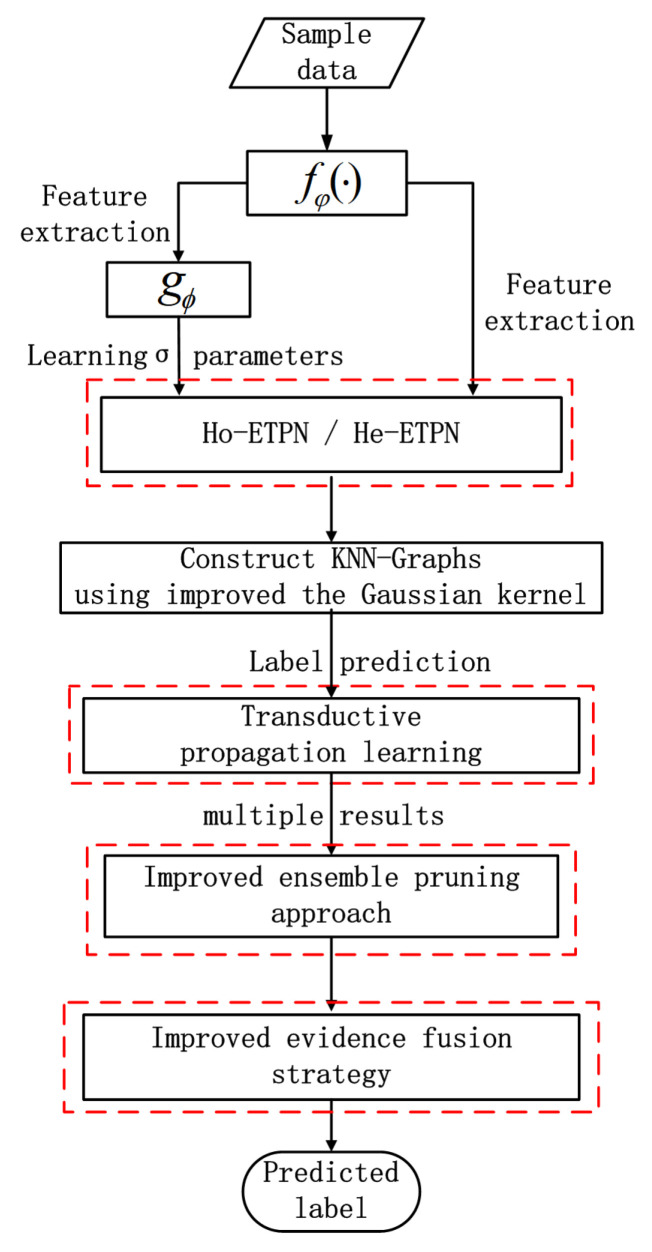
The overall flow chart of the ensemble model.

There are several important parts in our ETPN model (as shown in Figure 2), including the framework of Ho-ETPN and He-ETPN, constructing KNN-Graphs using the improved Gaussian kernel [41], transductive propagation learning, and evidence fusion strategy. Next, we introduce the single model framework of IG-semiTPN simply, then introduce other parts of our ensemble model in detail.

### 4.1. IG-semiTPN Model

**Figure 3:**
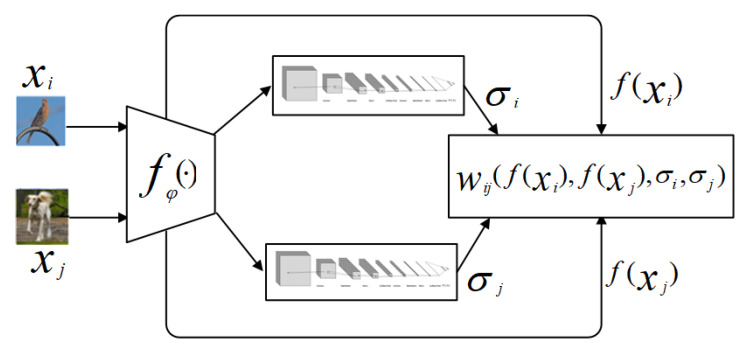
The framework of the IG-semiTPN model.

We propose our ensemble semi-supervised graph network based on the individual learner framework of IG-semiTPN [41] to utilize the information shared between support and query datasets. The framework of IG-semiTPN is shown in Figure 3.

**Figure 4:**
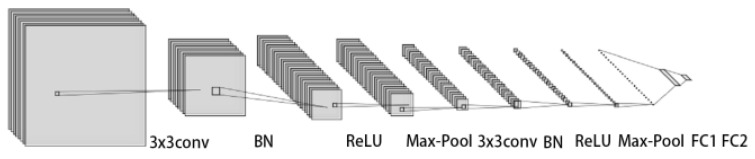
$g_{\phi }$ construction of model.

Firstly, it employs $f_{\varphi }\left(\cdot \right)$ to extract features of the input $x_{i}$ and $x_{j}$ (φ indicates a parameter of the network). Then, the graph construction module $g_{\phi }$ (as shown in Figure 4) is utilized to learn $\sigma_{i}$ for every instance. Next, an improved Gaussian kernel $w_{ij}\left(f_{\varphi }\left(x_{i}\right),f_{\varphi }\left(x_{j}\right),\sigma_{i},\sigma_{j}\right)$ is proposed to calculate the edge weight for constructing the k-nearest neighbor graph. Finally, the label propagation method is adopted to achieve transductive propagation learning.

### 4.2. ETPN Model

**Figure 5:**
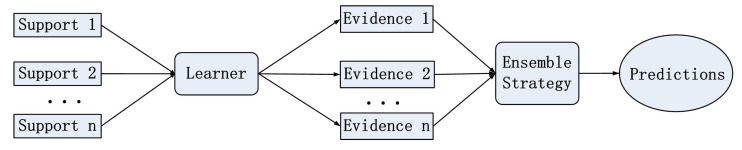
The process of homogeneous ensembles.

**Figure 6:**
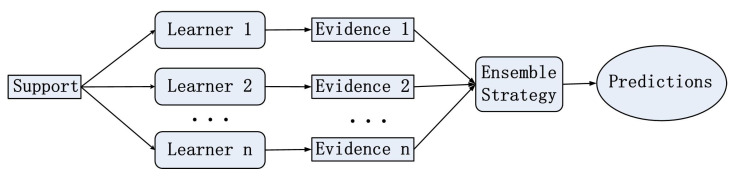
The process of heterogeneous ensembles.

Ensemble learning aims to enhance the generalization ability and stability of individual learners. The homogeneous framework employs a single base learning algorithm, i.e., learners of the same type but with multiple different sample inputs, leading to homogeneous ensembles (shown in Figure 5). The heterogeneous model utilizes multiple learning algorithms, i.e., learners of different types, leading to heterogeneous ensembles (shown in Figure 6).

#### 4.2.1. The Ho-ETPN Model

**Figure 7:**
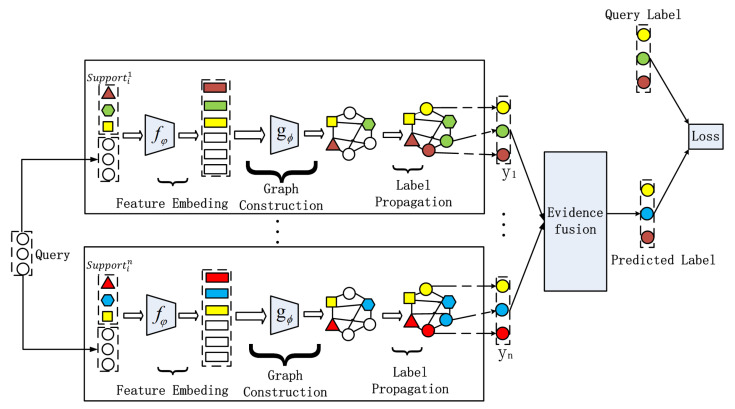
The framework of the Ho-ETPN model.

In this section, we propose a homogeneous ensemble few-shot learning model (Ho-ETPN, shown in Figure 7). The Ho-ETPN model generates multiple results (evidence) by randomly selecting different support sets (i.e., $support_{i}^{n}$, being the same categories but different samples) in every episodic training. In contrast, the query set is selected only once in each episodic training. It generates multiple results by the same individual learner introduced in the last sector then integrates them via the the evidence fusion strategy proposed in this paper to accomplish predictions.

#### 4.2.2. The He-ETPN Model

**Figure 8:**
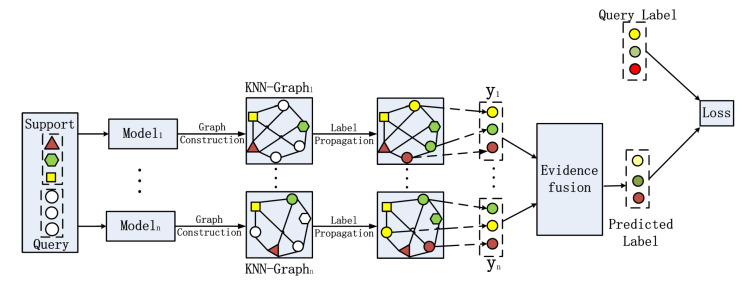
The framework of the He-ETPN model.

In this section, we propose a heterogeneous ensemble few-shot learning model (He-ETPN). The He-ETPN model generates multiple results (evidence) via multiple learners in every episodic training, and then integrates them via the evidence fusion strategy proposed in this paper to accomplish predictions. The model framework of base learners has been introduced in the last section. The He-ETPN model (shown in Figure 8) generates multiple results by constructing diverse KNN-graphs using different models that are the different initializations of $f_{\varphi }\left(\cdot \right)$ and $g_{\phi }$, with different value settings of γ and *m* in an improved Gaussian kernel.

#### 4.2.3. Construct KNN-Graphs

For dataset $S=S_{sup}\cup S_{uns}$, during the construction of KNN-graphs, let s∈S represent the graph vertex to build the undirected graphs of labeled and unlabeled samples. We use the edge weights to measure the similarity between samples, the greater the weight of the edge, the greater the similarity between the two samples. Due to the improved Gaussian kernel [41] the nuclear truncation effect is alleviated by adding displacement parameters and corrections and learning a σ parameter suitable for each sample in the process of constructing the graph. Therefore, we utilize the improved Gaussian kernel to calculate the edge weight to construct more accurate KNN graphs for the transductive propagation ensemble network. The improved Gaussian kernel is defined as follows:

(1) $$\begin{array}{c} w_{ij}=exp\left(\frac{1}{md\left(\frac{f_{\varphi }\left(x_{i}\right)}{\sigma_{i}},\frac{f_{\varphi }\left(x_{j}\right)}{\sigma_{j}},m\right)+\gamma^{2}}+\lambda \right),i,j=1,\ldots ..,n, \end{array}$$

where $f_{\varphi }\left(\cdot \right)$ refers to the feature map, φ indicates a parameter of the network, σ is the variable bandwidth (length scale parameter) of the kernel function learned by $g_{\phi }$, γ is the displacement parameters, λ is the fine-tuning variable. d(·) is the Euclidean distance.

### 4.3. Transductive Propagation Learning

**Figure 9:**
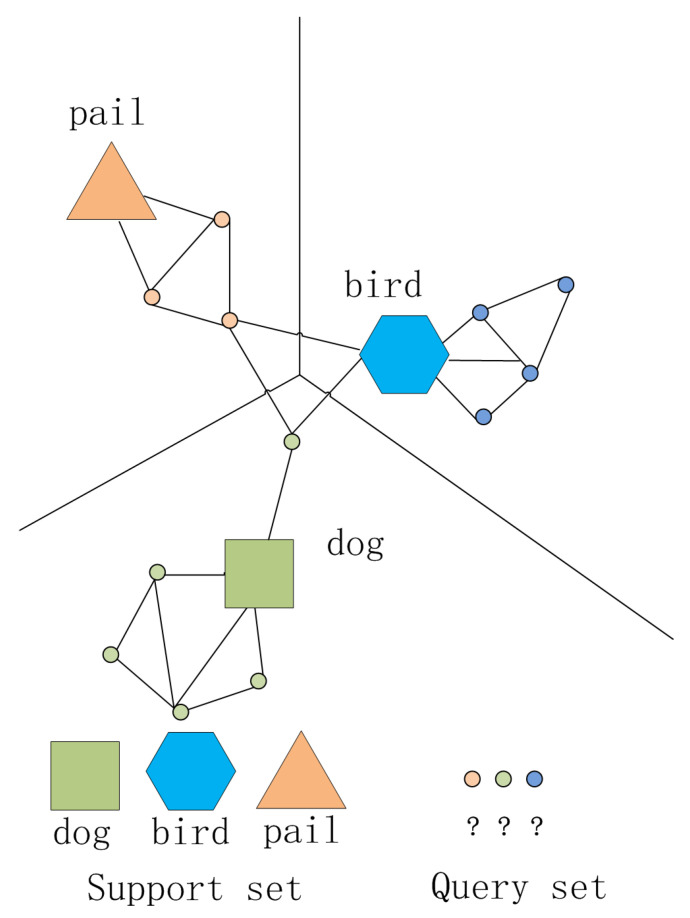
Transductive propagation algorithm based on K-nearest neighbor graph.

Transductive propagation learning aims to predict unlabeled data from locally labeled training samples. It takes the support and query set as graph nodes, then makes joint predictions using the nearest neighbor relationship between the support and query sets (as shown in Figure 9), which can supplement the deficiency of label information through unlabeled data. Due to its low complexity and good classification effect, the label propagation algorithm is adopted to perform the transfer of label information between graph nodes. The process of predictions for the query set $Q^{M}$ using label propagation is defined as follows:

(1) Suppose $F=[Y_{l},Y_{u}]$ is an annotation matrix with (K·N+M)×K−dimension. $Y_{l}$ denotes the support set sample label matrix, and $Y_{u}$ denotes the query set sample matrix Let *Y* is the initial annotation matrix Y∈F, $Y_{ic}$ represents the membership degree to the c−th column category of i−th node $Y_{i}$. If $Y_{ic}=1$, which is mean the node $Y_{i}$ belonging to the category *c*, else $Y_{ic}=0$, that is,

(2) $$Y_{ic}=\left\{\begin{array}{c} \begin{array}{cc} 1,\;\; & Y_{i}=c\\ 0,\;\; & otherwise \end{array} \end{array}\right.$$

(2) Given the initial label matrix $Y_{l}$, the query set labels are iteratively predicted according to the KNN-graphs. Let α∈(0,1) denote the pre-set weight coefficient to control the amount of propagated information. The transductive propagation learning iteratively inferences as follows:

(3) $$\begin{array}{cc} \; & \lim_{\begin{array}{c} x\to \infty \end{array}}T^{t}=\left(\begin{array}{cc} I & 0\\ \alpha \cdot T_{ul} & \alpha \cdot T_{uu} \end{array}\right)\times \left(\begin{array}{cc} I & 0\\ \alpha \cdot T_{ul} & \alpha \cdot T_{uu} \end{array}\right)\times \left(\begin{array}{cc} I & 0\\ \alpha \cdot T_{ul} & \alpha \cdot T_{uu} \end{array}\right)\times \ldots \\ \; & =\left(\begin{array}{cc} I\cdot I+0+0+\ldots & 0+0+0+\ldots \\ \left(\alpha \cdot T_{ul}\right)+\left(\alpha \cdot T_{ul}\right)\cdot \left(\alpha \cdot T_{uu}\right)+\left(\alpha \cdot T_{ul}\right)\cdot {\left(\alpha \cdot T_{uu}\right)}^{2}+\ldots & \left(\alpha \cdot T_{uu}\right)\cdot \left(\alpha \cdot T_{uu}\right)\cdot \left(\alpha \cdot T_{uu}\right)\ldots \end{array}\right)\\ \; & =\left(\begin{array}{cc} I\cdot I+0+0+\ldots & 0+0+0+\ldots \\ \left(I+\left(\alpha \cdot T_{uu}\right)+{\left(\alpha \cdot T_{uu}\right)}^{2}+\ldots \right)\cdot \left(\alpha \cdot T_{ul}\right) & \left(\alpha \cdot T_{uu}\right)\cdot \left(\alpha \cdot T_{uu}\right)\cdot \left(\alpha \cdot T_{uu}\right)\ldots \end{array}\right)\\ \; & =\left(\begin{array}{cc} I & 0\\ \left(\sum_{t=0}^{\infty }{\left(\alpha \cdot T_{uu}\right)}^{t}\right)\cdot \left(\alpha \cdot T_{ul}\right) & {\left(\alpha \cdot T_{uu}\right)}^{\infty } \end{array}\right)\\ \; & =\left(\begin{array}{cc} I & 0\\ {\left(I-\alpha T_{uu}\right)}^{-1}\cdot \left(\alpha \cdot T_{ul}\right) & 0 \end{array}\right) \end{array}$$

(4) $$\begin{array}{c} F_{t+1}=\lim_{\begin{array}{c} x\to \infty \end{array}}F_{t}=\lim_{t\to \infty }\;T^{t}\left(\begin{array}{c} \left(1-\alpha \right)Y_{l}\\ 0 \end{array}\right)=\left(\begin{array}{cc} I & 0\\ {\left(I-\alpha T_{uu}\right)}^{-1}\cdot \left(\alpha \cdot T_{ul}\right) & 0 \end{array}\right)\left(\begin{array}{c} \left(1-\alpha \right)Y_{l}\\ 0 \end{array}\right), \end{array}$$

where $T=D^{-1/2}WD^{-1/2}$, *T* represents the normalized Laplace matrix. $T_{ll}$ denotes the identity matrix; $T_{lu}$ denotes the zero matrix; $T_{ul}\subset T$; $T_{uu}\subset T$. $W=[w_{ij}]$, $W\in R^{\left(K\cdot N+M)\times (K\cdot N+M\right)}$ for all instances in $S=S_{sup}\cup S_{uns}$.

We keep the k−max values in each row of *W* calculating by Equation (1) to construct KNN-graphs. Then, the normalized graph Laplacian is applied [44] on *W*; *D* is the diagonal matrix, $D=diag(\left[d_{ii}=\sum_{j=1}^{K\cdot N+M}w_{ij}\right])$. While α is bigger, the results tend to favor label propagation items $T_{uu}$ and $T_{ul}$, else, results prefer the original annotated items $Y_{l}$. The final prediction results $F^{*}$( $F^{*}=\left[f_{i\eta }\right]$) are obtained through multiple iterations, as is shown in Equation (5).

(5) $$\begin{array}{c} F^{*}=\lim_{\begin{array}{c} x\to \infty \end{array}}F_{t}={\left(I-\alpha T_{uu}\right)}^{-1}\left(\alpha \cdot T_{ul}\right)\cdot \left(1-\alpha \right)Y_{l}. \end{array}$$

### 4.4. Ensemble Pruning

The error-ambiguity decomposition [45] can show that the success of ensemble learning depends on a good trade off between the individual performance and diversity, which is defined as follows:

(6) $$\begin{array}{c} err\left(H\right)=\bar{err}\left(h\right)-\sqrt{ambi}\left(h\right), \end{array}$$

where $\bar{err}\left(h\right)=\sum_{i=1}^{N}\varpi_{i}\cdot err\left(h_{i}\right)$ denotes the average error of the individual learners, and $\bar{err}\left(h\right)=\sum_{i=1}^{N}\varpi_{i}\cdot ambi\left(h_{i}\right)$ denotes the weighted average of ambiguities. *h* denotes the individual learner.

The $\bar{err}\left(h\right)$ depends on the generalization ability of individual learners; the $\bar{ambi}\left(h\right)$ depends on the ensemble diversity. Since the $\bar{ambi}\left(h\right)$ is always positive, obviously, the error of the ensemble will never be larger than the average error of the individual learners. More importantly, Equation (6) shows that the more accurate and the more diverse the individual learners, the better the ensemble. Based on this, we propose an improved ensemble pruning approach to select more accurate learners to participate in the integration.

Ensemble pruning is to associate an individual learner with a weight that could characterize the goodness of including the individual learner in the final ensemble. RSE is a regularized selective ensemble algorithm; it adopted the L1 norm for feature selection to obtain sparse weights [46]. However, the sample space of few-shot learning is more sparse. To enhance data utilization and ensure that samples far away from decision boundaries still contribute to model training, we employ the L2 norm to obtain weights as small as possible but not zero. In addition, this makes the model more stable to small changes of the input and improves the robustness of the model. Moreover, to be suitable for few-shot learning, we redefine the improved ensemble pruning algorithm.

Given *n* individual learners for He-ETPN or Ho-ETPN, let $\varpi =[\varpi_{1},\ldots ,\varpi_{n}]$ denote the n-dimensional weight vector of *n* individual learners, where small elements in the weight vector suggest that the corresponding individual learner from He-ETPN or Ho-ETPN should be excluded during the process of fusion.

(7) $$\begin{array}{c} \Theta (\varpi )=\lambda \Lambda (\varpi )+\Omega (\varpi ), \end{array}$$

where Λ(ϖ) is the empirical loss, Ω(ϖ) is the graph Laplacian regularization term to measure the misclassification, and λ is a regularization parameter which trades off the minimization of Λ(ϖ) and Ω(ϖ).

By introducing slack variables η and minimizing the regularized risk function to determine the weight vector, Equation (7) is redefined as follows:

(8) $$\begin{array}{cc} \; & \min_{\varpi }\varpi P^{⊤}TP\varpi^{⊤}+\lambda \left(\eta_{1}^{2}+\ldots +\eta_{m}^{2}\right)\\ \; & s.t.\;\;y_{i}p_{i}\varpi^{⊤}+\eta_{i}^{2}\geq 1,\left(\forall i=1,\ldots ,m\right)\\ \; & \varpi_{1}+\ldots +\varpi_{m}=1,\varpi \geq 0, \end{array}$$

where *P* denotes the prediction matrix of all individual learners on all support set instances, $p_{i}=\left(\max\left(F_{1}^{*}\right),\ldots ,\max\left(F_{n}^{*}\right)\right)$ denotes the predictions of the individual learner on $x_{i}$, and *T* represents the normalized Laplace matrix. $y_{i}$ denotes the sample label of $x_{i}$. $\eta ={\left(\eta_{1},\ldots ,\eta_{m}\right)}^{⊤}$ denotes the slack variables.

(9) $$\varpi_{i}=\left\{\begin{array}{c} \begin{array}{cc} 1,\;\; & top-n\\ 0,\;\; & otherwise \end{array} \end{array}\right.$$

(10) $$\begin{array}{c} F_{i}^{*}=\varpi_{i}\cdot F_{i}^{*},i=1,\ldots ,n, \end{array}$$

where top−n is to select the top *n* best individual learners for the pruned ensemble. $F_{i}^{*}$ denotes a piece of evidence, if the $\varpi_{i}=0$ denotes that the $F_{i}^{*}$ does not participate in the fusion of the results.

The complexity of the pruning approach is $O(n^{3})$. Equation (8) is a standard QP problem that can be efficiently solved by existing optimization packages. It is more suitable for small-scale datasets, especially few-shot learning.

### 4.5. Evidence Fusion Strategy

In this paper, we propose an improved D-S evidence fusion method to assemble the multiple pieces of evidence generated by the ensemble solutions of the Ho-ETPN and He-ETPN. Compared with the averaging and voting methods, the improved D-S evidence fusion method can enhance the stability of ensemble results and alleviate the problem of the “Zadeh paradox” to a certain extent. The D-S evidence theory was first proposed by Dempster [47,48]. Combining multiple information sources is an effective method of uncertainty reasoning. The research indicates that the synthetic consequence of conventional combination rules of Dempster is frequently contrary to the reality in the practical applications [49,50]. Two major approaches are proposed to improve the accuracy of synthetic results—one is to amend the composition rules; the second is to change the original evidence resources. In this paper, we focus on the latter. Next, we concretely introduce the process of the improved D-S evidence fusion method.

(1) Conflict Matrix

The Bhattacharyya distance [51] is utilized to construct the conflict matrix between evidence. According to the intension of Bhattacharyya distance, the formula is redefined as follows:

**Definition** **1** (Bhattacharyya Distance). For probability distributions $F_{i}^{*}$ and $F_{j}^{*}$ over the same domain, the Bhattacharyya distance is defined as:

(11) $$\begin{array}{c} Dis_{BC}\left(F_{i}^{*},F_{j}^{*}\right)=\sum_{\eta =1}^{K}\sqrt{F_{i}^{*}\left(f_{i\eta }\right)F_{j}^{*}\left(f_{j\eta }\right))} \end{array}$$

(12) $$\begin{array}{c} fc_{ij}=-ln\left(Dis_{BC}\left(F_{i}^{*},F_{j}^{*}\right)\right), \end{array}$$

where $Dis_{BC}\left(F_{i}^{*},F_{j}^{*}\right)$ is the Bhattacharyya coefficient for discrete probability distributions. Let n=K·N+M denote the number of pieces of evidence. Each piece of normalized evidence is denoted by $F_{i}^{*}=\left(f_{i1},f_{i2},\ldots ,f_{iK}\right)$. $F_{i}^{*}$ and $F_{j}^{*}\left(1\leq i,j\leq n\right)$ represent two pieces of evidence. The K denotes the number of classes in each support set, $k_{i}\in K$. Then, the normalization conflict matrix is defined as:

(13) $$\begin{array}{c} Matrix_{conflict}=\left[\begin{array}{cccccc} 0 & fc_{12} & \cdots & fc_{1j} & \cdots & fc_{1n}\\ fc_{21} & 0 & \cdots & fc_{2j} & \cdots & fc_{2n}\\ \vdots & \vdots & \vdots & \vdots & \vdots & \vdots \\ fc_{j1} & fc_{j2} & \cdots & 0 & \cdots & fc_{jn}\\ \vdots & \vdots & \vdots & \vdots & \vdots & \vdots \\ fc_{n1} & fc_{n2} & \cdots & fc_{nj} & \cdots & 0 \end{array}\right]. \end{array}$$

(2) Support Degree

Evidence support degree indicates the support degree of evidence that is supported by other evidence. The higher the similarity with other evidence, the higher the support degree it is, and vice versa. According to $Matrix_{conflit}$ the following formula is utilized to calculate the similarity degree between $F_{i}^{*}$ and $F_{j}^{*}$.

(14) $$\begin{array}{c} sd_{ij}=1-fc_{ij},\;\;\;i,j=1,2,\ldots ,n. \end{array}$$

As a result, we can obtain the following similarity matrix of all evidence:

(15) $$\begin{array}{c} Matrix_{similarity}=\left[\begin{array}{cccccc} 1 & sd_{12} & \cdots & sd_{1j} & \cdots & sd_{1n}\\ sd_{21} & 1 & \cdots & sd_{2j} & \cdots & sd_{2n}\\ \vdots & \vdots & \vdots & \vdots & \vdots & \vdots \\ sd_{j1} & sd_{j2} & \cdots & 1 & \cdots & sd_{jn}\\ \vdots & \vdots & \vdots & \vdots & \vdots & \vdots \\ sd_{n1} & sd_{n2} & \cdots & sd_{nj} & \cdots & 1 \end{array}\right]. \end{array}$$

And then, the support degree of each evident is calculated as:

(16) $$\begin{array}{c} Sup\left(F_{i}^{*}\right)=\sum_{j=1,j\neq i}^{n}sd_{ij}. \end{array}$$

(3) Evident Weight

Credibility degree indicates the credibility of an evidence. It can be calculated by following formula.

(17) $$\begin{array}{c} CR\left(F_{i}^{*}\right)=\frac{Sup(F_{i}^{*})}{\sum_{j=1}^{n}Sup\left(F_{j}^{*}\right)}. \end{array}$$

Information entropy can be utilized to measure the informative quantity of evidence in the information fusion process. Integrated with D-S evidence theory, given a piece of evidence $F_{i}^{*}=\left(f_{i1}f{,}_{i2},\ldots ,f_{iK}\right)$, and $\sum_{\eta =1}^{K}f_{i\eta }=1$. The information quantity of the ith piece of evidence is defined as:

(18) $$\begin{array}{c} Info_{e}\left(F_{i}^{*}\right)=-\sum_{\eta =1}^{K}f_{i\eta }logf_{i\eta }. \end{array}$$

For information entropy, the larger the uncertainty, the smaller its weight. On the other hand, the smaller the information entropy, the larger its weight. The method mentioned above can be used to reduce the weight ratio of the evidence with higher indeterminacy in the fusion process. Therefore, the weight of each evidence is defined as:

(19) $$\begin{array}{c} weight\left(F_{i}^{*}\right)=\frac{CR(F_{i}^{*})}{Normalization(Info_{e}\left(F_{i}^{*}\right))},\;1\leq i\leq n \end{array}$$

(20) $$\begin{array}{c} F_{i}^{*}=weight\left(F_{i}^{*}\right)\times F_{i}^{*}. \end{array}$$

(4) Evidence Combination Rule

Suppose that the feature subsets generated in the previous chapter are independent. The D-S evidence theory improved in this paper allows the fusion of information coming from different feature subsets. Therefore, the evidence combination rule is utilized to combine different weighted feature subsets in a manner that is both accurate and robustness.

For $F_{i}^{*}\left(i=1,2,\ldots ,n\right)$, ∀k∈K, the combination rule is redefined as:

(21) $$\begin{array}{c} F_{fusion}^{*}=\left(F_{1}^{*}\;⊎\;F_{2}^{*}\;⊎\cdots ⊎\;F_{n}^{*}\right)\left(k\right)=\frac{1}{Q}\prod_{i=1}^{n}F_{i}^{*}\left(k\right), \end{array}$$

where *Q* means the conflict between different pieces of evidence, is given by:

(22) $$\begin{array}{c} Q=\sum_{i=1}^{K}\prod_{j=1}^{n}F_{j}^{*}\left(k_{i}\right). \end{array}$$

### 4.6. Loss Generation

In this paper, we adopt cross-entropy loss to calculate the similarity between predictive values and true values.

(1) We adopt the softmax function to transform the $F_{fusion}^{*}$ of the ETPN model to probability, which is defined as follows:

(23) $$\begin{array}{c} P\left(\tilde{y_{i}}=j|x_{i}\right)=\frac{exp(f_{i\eta })}{\sum_{j=1}^{N}exp\left(f_{i\eta }\right)}, \end{array}$$

where $\tilde{y_{i}}$ is the final prediction value of i−th samples in query. $f_{i\eta }$ is the component of the prediction values in label propagation.

(2) We calculate the loss by the cross entropy loss:

(24) $$\begin{array}{c} J\left(\varphi ,\phi \right)=\sum_{i=1}^{T}\sum_{j=1}^{N}-I\left(y_{i}=j\right)log\left(P\left(\tilde{y_{i}}=j|x_{i}\right)\right), \end{array}$$

where $y_{i}$ is the true value of the instance. I(b) is the indicator function. If *b* is right, and I(b)=1, else I(b)=0.

## 5. Experiments

In this section, to validate the performance of models, we contrast our proposal against state-of-the-art techniques on miniImageNet and tieredImageNet datasets. In addition, we set a supervised experiment including the ensemble model Ho-ETPN and He-ETPN, and a semi-supervised experiment including the setting of distractor classes. These approaches are particularly divided into optimization-based (MAML [22]), ensemble-based (EBDM-Euc [38], HGNN [39], E^3^BM + MAML [40]), graph-based (TPN [25], EPNet [27], TPRN [31], DSN [32], EGNN [33], PRWN [35], GNN [52], BGNN^*^ [53], DPGN^*^ [54]), and metric-based (MatchingNet [8], Proto Net [9], TADAM [13], BR-ProtoNet [36], SSFormers [55], CGRN [56], HMRN [57]) approaches. Moreover, we conduct 5-way 1-shot and 5-shot experiments, which are standard few-shot learning settings.

### 5.1. Datasets

miniImageNet [8]. A subset of the ImageNet datasets [58] consists of 60,000 images. Each image is of size 84 × 84, and classes with 600 samples per class are divided into 64, 16, and 20 for meta-training, meta-validation, and meta-testing, respectively. We use the miniImageNet for semi-supervised classification with 40% of labeled data.

tieredImageNet [41]. A more challenging subset derived from ImageNet datasets, its class subsets are chosen from supersets of the wordnet hierarchy. The top hierarchy has 34 super-classes, which are split into 20 different categories (351 classes) for training, six different categories (97 classes) for validation, and eight different categories (160 classes ) for testing. We follow the implementation of 4-convolutional layer (Conv−4) backbones and the image size of 84 × 84 as on miniImageNet. Moreover, the tieredImageNet is used for semi-supervised classification with 10% of labeled data.

### 5.2. Implementation Details

Following the Matching Networks [8], we also adopt the episodic training procedure. Moreover, we used a common feature extractor, which is a Conv−4 as implemented in [8] during the entire comparision experiments for standard few-shot classification. It makes up four convolutional blocks where each block begins with a 2D convolutional layer with a 3 × 3 kernel and a filter size of 64. Each convolutional layer is followed by a batch-normalization layer [43], a ReLU nonlinearity, and a 2 × 2 max-pooling layer. Moreover, $g_{\phi }$ utilized to learn $\sigma_{i}$ for every instances, consists of two convolutional blocks (64 and 1 filters) and two fully-connected layers (8 and 1 neurons) similar to TPN [25]. The convolutional blocks are made up of four convolutional blocks and each block begins with a 2D convolutional layer with a 3 × 3 kernel and filter size of 64. Each convolutional layer is followed by a batch-normalization layer [43], a ReLU nonlinearity and a 2 × 2 max-pooling layer. In the experiments, we follow a general practice to evaluate the model with N-way K-shot and 15 query images; the value of λ is set to 0.75. And we use Adam optimizer [59] with an initial learning rate of 0.001, we use the validation set to select the training episodes with the best accuracy, and run the training process until the validation loss reaches a plateau.

In addition, we utilize the improved Gaussian kernel proposed in the single model framework IG-semiTPN to construct the KNN graphs. IG-semiTPN experiments showed the superior effects of the improved Gaussian kernel function. It also indicated that the optimal models have relations with the value of γ [60,61]. Therefore, ETPN utilizes the parameter settings of the improved Gaussian kernel of the IG-semiTPN to perform supervised and semi-supervised experiments. Specifically, Ho-ETPN adopts the Minkowski distance with γ being 3 and *m* being 3 or the Minkowski distance with *m* being 2 and λ is 0.75. In addition, there are three learners in our He-ETPN ensemble models; learner 1 adopts a Minkowski distance with γ being 3 and *m* being 3; learner 2 adopts a Minkowski distance with γ being 0.2 and *m* being 2; learner 3 adopts a Minkowski distance with *m* being 2, and λ is 0.75.

### 5.3. Supervised Experiment

#### ETPN Experiment

**Figure 10:**
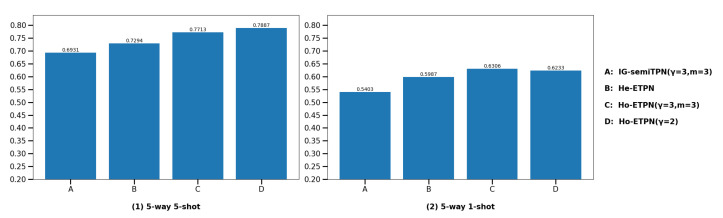
Comparison of the IG-semiTPN and ETPN (He-ETPN and Ho-ETPN) on miniImageNet.

**Figure 11:**
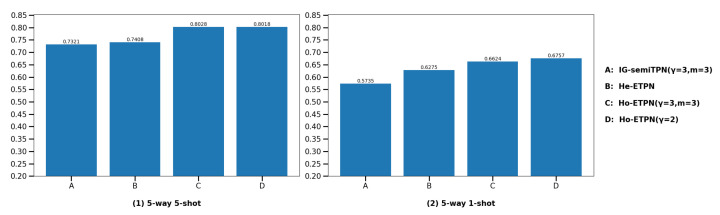
Comparison of the IG-semiTPN and ETPN (He-ETPN and Ho-ETPN) on tieredImageNet.

In our experiments, we compare ensemble model ETPN with other classic and advanced algorithms in four categories, including graph-based (TPN [25], EPNet [27], TPRN [31], DSN [32], EGNN [33], PRWN [35], GNN [52], BGNN^*^ [53], DPGN^*^ [54]), metric-based (MatchingNet [8], Proto Net [9], TADAM [13], BR-ProtoNet [36], SSFormers [55], CGRN [56], HMRN [57]), optimization-based (MAML [22]) and ensemble-based (EBDM-Euc [38], HGNN [39], E^3^BM + MAML [40]) approaches. The performance of the proposed ETPN and state-of-the-art models in the 5-way 5-shot/1-shot accuracy on the miniImageNet and tieredImageNet datasets are summarized in Table 2 and Table 3, and "*" in the table indicates results re-implemented in HGNN [39] for a fair comparison. Our proposed ETPN outperforms few-shot models by large margins, indicating that the proposed ensemble model effectively assists few-shot recognition. Specifically, we can obtain the following observations:

(1) **Comparison with the latest model.** Ho-ETPN is 8.32% higher than SSFormers in 5-shot on miniImageNet and 5.58% higher than HMRN in 5-shot on tieredImageNet; Ho-ETPN is 7.86% higher than SSFormers in 1-shot on miniImageNet and 9.59% higher than HMRN in 1-shot on tieredImageNet. It confirms that our model has a good ability for classification discrimination, which benefits from our ensemble model and D-S evidence fusion strategy based on improved ensemble pruning.

(2) **Comparison with the state-of-the-art.** Under the 5-way-5-shot setting, the ETPN classification accuracies are 78.87% vs. 78.57% for the transductive learning model TPRN, 80.28% vs. 80.0% ensemble model BR-ProtoNet on miniImageNet and tieredImageNet, respectively. It is 0.3% higher than the transductive learning model TPRN in 5-shot on miniImageNet and 0.28% higher than the ensemble model BR-ProtoNet in 5-shot on tieredImageNet. Under the 5-way-1-shot setting, the ETPN classification accuracies are 63.06% vs. 57.84% for the transductive learning model TPRN, 67.57% vs. 62.7% for the ensemble model BR-ProtoNet on miniImageNet and tieredImageNet, respectively. It is 5.22% higher than the transductive model TPRN in 1-shot on miniImageNet and 4.87% higher than the ensemble model BR-ProtoNet in 1-shot on tieredImageNet. ETPN outperforms state-of-the-art few-shot models by large margins, especially under the 5-way-1-shot setting. This indicates that our ensemble model and improved evidence fusion strategy are effective, particularly for scenarios with a small sample size, which can increase the performance by enhancing the stability of the model.

(3) **Compare with individual learner IG-semiTPN.** In this section, we compare our ensemble model ETPN with our single supervised model IG-semiTPN [41]; this is to show that the homogeneous strategy, heterogeneous strategy, and improved D-S evidence fusion strategy based on improved ensemble pruning facilitate the model performance. For the fairness of the experiment, we ensure other settings are the same, only changing the ensemble strategy and the parameter settings in the improved Gaussian kernel to perform the ablation experiment. The comparison results are shown in Figure 10 and Figure 11. Under the 5-way 5-shot setting, the classification accuracies of Ho-ETPN and IG-semiTPN are 78.87% vs. 69.31% on miniImageNet, and 80.28% vs. 73.21% on tieredImageNet, respectively. Ho-ETPN is 9.56% and 7.07% higher than IG-semiTPN in 5-shot on miniImageNet and tieredImageNet, respectively. Under the 5-way 1-shot setting, the classification accuracies of Ho-ETPN and IG-semiTPN are 63.06% vs. 54.03% on miniImageNet, and 67.57% vs. 57.35% on tieredImageNet, respectively. Ho-ETPN is 9.03% and 10.22% higher than IG-semiTPN in 1-shot on miniImageNet and tieredImageNet, respectively. In addition, under the 5-way 5-shot setting, the classification accuracies of He-ETPN and IG-semiTPN are 72.94% vs. 69.31% on miniImageNet, and 74.08% vs. 73.21% on tieredImageNet, respectively. He-ETPN is 3.63% and 0.87% higher than IG-semiTPN in 5-shot on miniImageNet and tieredImageNet, respectively. Under the 5-way 1-shot setting, the classification accuracies of He-ETPN and IG-semiTPN are 59.87% vs. 54.03% on miniImageNet, and 62.75% vs. 57.35% on tieredImageNet, respectively. He-ETPN is 5.84% and 5.4% higher than IG-semiTPN in 1-shot on miniImageNet and tieredImageNet, respectively. The results indicate the effectiveness of the proposed ensemble solutions, which achieve a state-of-the-art performance compared to the single model IG-semiTPN, especially in 1-shot. Moreover, the Ho-ETPN is superior to the He-ETPN, which is related to the problem of multiple learner selection. In our paper, we only select different parameter settings of $f_{\varphi }$, $g_{\phi }$ and an improved Gaussian kernel.

### 5.4. Semi-Supervised Experiment

Since labeled data are scarce and their collection is expensive, in this section, we leverage the extra unlabeled data to improve the performance of few-shot classifiers. Our model was trained on miniImageNet and tieredImageNet with 40% and 10% of labeled data, respectively. What is more, another key challenge is that the distractor classes, being an unlabeled set that is irrelevant to the classification task, are introduced to boost robustness against perturbations. We follow the settings in papers [41,63]. Our models outperforms inference (TADAM-semi [13], BR-ProtoNet [36] and PN+Semi [41]) and transduction (TPN-semi [25], Semi-EPNet [27], Semi DSN [32], Semi-EGNN [33] and PRWN-semi [35]) semi-supervised few-shot models by large margins.

**Figure 12:**
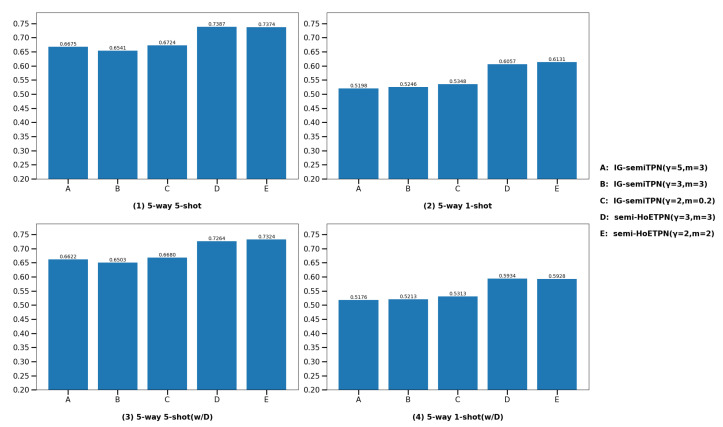
Comparison of the IG-semiTPN and semi-HoETPN on miniImageNet.

**Figure 13:**
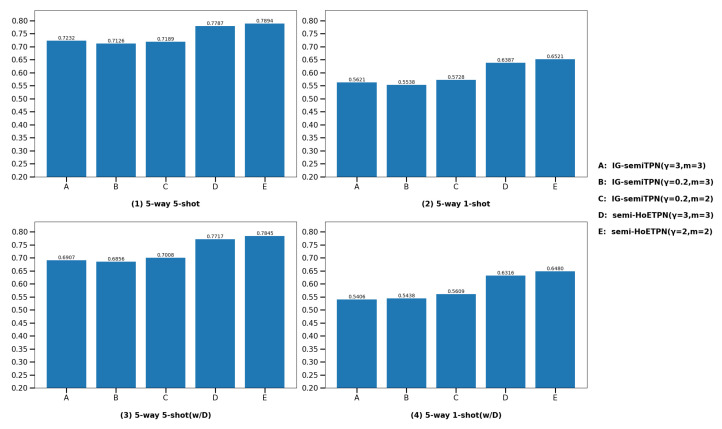
Comparison of the IG-semiTPN and semi-HoETPN on tieredImageNet.

(1) **Comparison with the state-of-the-art.** In order to ensure the effectiveness of the semi-supervised experiment, every category in the datasets was divided into labeled datasets and unlabeled datasets without intersection [39]. In this paper, we utilize the label propagation algorithm to perform the annotation for unlabeled data, which is different from traditional inductive reasoning semi-supervised approaches. As is shown in Table 4 and Table 5, it can be observed that the classification results of all semi-supervised few-shot models are degraded due to the distractor classes. However, even with the distractor class represented as w/D in the table, the ensemble semi-supervised model semi-HoTPN achieves the highest performance among the compared methods, especially in the scenario of 1-shot, which indicates the robustness of the proposed semi-HoTPN in dealing with distracted unlabeled data. In addition, this indicates that the proposed D-S evidence fusion strategy based on improved ensemble pruning, transductive propagation learning and homogeneous ensemble semi-supervised model semi-HoTPN effectively assists few-shot recognition.

(2) **Compare with individual learner IG-semiTPN.** In this section, we show that the semi-supervised homogeneous ensemble model and improved D-S evidence fusion strategy based on improved ensemble pruning facilitate the model performance. For the fairness of the experiment, we compare semi-HoETPN with IG-semiTPN and other settings are the same. The comparison results are shown in Figure 12 and Figure 13. We compare ensemble semi-supervised model semi-HoETPN with single semi-supervised model IG-semiTPN; under the 5-way-5-shot setting, the classification accuracies of semi-HoTPN and IG-semiTPN are 73.87% vs. 67.24% on miniImageNet, and 78.94% vs. 72.32% on tieredImageNet, respectively. Under the 5-way-1-shot setting, the classification accuracies of semi-HoTPN and IG-semiTPN are 61.31% and 53.48% on miniImageNet, and 65.21% and 57.28% on tieredImageNet, respectively. With the distractor class experiments, under the 5-way-5-shot setting, the classification accuracies of semi-HoTPN and IG-semiTPN are 73.24% vs. 66.8% on miniImageNet, and 78.45% vs. 70.08% on tieredImageNet, respectively; under the 5-way-1-shot setting, the classification accuracies of semi-HoTPN and IG-semiTPN are 59.34% vs. 53.13% on miniImageNet, and 64.80% vs. 56.09% on tieredImageNet, respectively. The results demonstrate the superior capacity of the proposed ensemble strategy in using the extra unlabeled information for boosting few-shot methods. Moreover, the addition of the distractor class enhances the robustness of the model.

## 6. Conclusions and Future Work

Few-shot learning aims to construct a classification model using limited samples. In this paper, we propose a novel ensemble semi-supervised few-shot learning with a transductive propagation network and evidence fusion. During the process of transductive propagation learning, we introduce the preset weight coefficient and calculate the process of iterative inferences to present homogeneous and heterogeneous models to improve the stability of the model. Then, we propose the improved D-S evidence ensemble strategy to enhance the stability of the final results. It combines the information entropy to realize the pre-processing of the evidence source. Then, an improved ensemble pruning method adopting the L2 norm is proposed to maintain a better performance of individual learners to enhance the accuracy of model fusion. Furthermore, an interference set is introduced to improve the robustness of the semi-supervised model. Experiments on miniImagnet and tieredImageNet indicate that the proposed approaches outperform the state-of-the-art few-shot model. However, our proposal directly utilizes a label propagation approach to transfer information between nodes in the graph-constructing phase. Therefore, in our future work, we will consider adopting the reality-semantic and cross-modal information to improve the accuracy of the transduction inference graph in few-shot learning.

## Figures and Tables

**Table 1:** The key contributions. Where ”w/D” means with distractors.

Model	trans_inference	SSFSL	Ensemble	EnsemblePruning	infor_uncertainty
TPRN [31]	✓	×	×	×	×
DSN [32]	✓	✓ (w/D)	×	×	×
PTN [26]	✓	✓ (w/D)	×	×	×
EGNN [33]	✓	✓	×	×	×
LTTL [34]	×	✓	×	×	×
PRWN [35]	×	✓ (w/D)	×	×	×
BR−ProtoNet [36]	×	✓ (w/D)	×	×	×
DIVERSITY [37]	×	×	✓	×	×
EBDM [38]	×	×	✓	×	×
HGNN [39]	✓	×	✓	×	×
$E^{3}BM$ [40]	×	×	✓	×	×
IG−semiTPN [41]	✓	✓ (w/D)	×	×	×
He−ETPN(our)	✓	✓ (w/D)	✓	✓	✓
Ho−ETPN(our)	✓	✓ (w/D)	✓	✓	✓

**Table 2:** Few-shot classification accuracies on the miniImageNet dataset are cited.

Methods	*m*	γ	λ	5way5shot	5way1shot
MatchingNet [8]				55.31%	43.56%
Proto Net [9]				68.20%	49.42%
TADAM [13]				76.7%	58.5%
BR-ProtoNet [36]				74.5%	58.4%
Relation Network [62]				65.32%	50.44%
MAML [22]				63.11%	48.70%
PRWN [35]				67.82%	50.89%
TPN [25]				67.79%	53.42%
EPNet [27]				72.95%	59.32%
TPRN [31]				78.57%	57.84%
DSN [32]				68.99 %	51.78%
EGNN [33]				66.85%	59.63%
GNN [52]				66.41%	50.33%
BGNN^*^ [53]				67.35	52.35%
DPGN^*^ [54]				65.34	53.22%
EBDM-Euc (2heads) [38]				68.30%	49.96%
EBDM-Euc (3heads) [38]				69.14%	50.49%
EBDM-Euc (5heads) [38]				69.64%	52.53%
EBDM-DD (2heads) [38]				67.99%	51.42%
EBDM-DD (3heads) [38]				68.74%	52.56%
EBDM-DD (5heads) [38]				70.17%	53.08%
HGNN [39]				72.48%	55.63%
E^3^BM + MAML [40]				65.1%	53.2%
SSFormers [55]				70.55%	55.2%
CGRN [56]				64.13%	50.85%
He-ETPN (our)			0.75	72.94%	59.87%
Ho-ETPN (our)	3	3	0.75	77.13%	**63.06**%
Ho-ETPN (our)	2		0.75	**78.87%**	62.33%

**Table 3:** Few-shot classification accuracies on the tieredImageNet dataset are cited.

Methods	*m*	γ	λ	5way5shot	5way1shot
Proto Net [9]				72.69%	53.31%
MAML [22]				70.30%	51.67%
PRWN [35]				70.52%	54.87%
BR-ProtoNet [36]				80.0%	62.7%
Relation Network [62]				71.32%	54.48%
TPN [25]				71.2%	56.17%
EGNN [33]				70.96%	
TPRN [31]				79.66%	59.26%
HGNN [39]				72.82%	56.05%
BGNN^*^ [53]				65.27%	49.41%
DPGN^*^ [54]				69.86%	53.99%
EPNet [27]				73.91%	59.97%
EBDM-Euc (3 heads) [38]				72.24%	51.22%
EBDM-Euc (1-st head) [38]				71.07%	50.04%
EBDM-Euc (2-nd head) [38]				71.28%	50.29%
EBDM-Euc (3-rd head) [38]				70.84%	50.52%
E^3^BM + MAML [40]				70.2%	52.1%
SSFormers [55]				73.72%	55.54%
CGRN [56]				71.34%	55.07%
HMRN [57]				74.70%	57.98%
He-ETPN (our)			0.75	74.08%	62.75%
Ho-ETPN (our)	3	3	0.75	**80.28%**	66.24%
Ho-ETPN (our)	2		0.75	80.18%	**67.57%**

**Table 4:** Semi-supervised comparison on the miniImageNet dataset.

Models	*m*	γ	λ	5way5shot	5way1shot	5way5shot (w/D)	5way1shot (w/D)
PN + Semi [41]				63.77%	49.98%	62.62%	47.42%
Soft k-Means [41]				64.59%	50.09%	63.55%	48.70%
Soft k-Means + Cluster [41]				63.08%	49.03%	61.27%	48.86%
Masked Soft k-Means [41]				64.39%	50.41%	62.96%	49.04%
TADAM-semi [13]						68.92%	54.81%
BR-ProtoNet [36]				73.1%	57.4%	72.4%	55.9%
Semi-EPNet [27]				67.08%			
Semi DSN [32]						67.12%	51.01%
Semi-EGNN [33]				64.32%			
PRWN-semi [35]				69.65%	56.65%	67.45%	53.61%
TPN-semi [25]				64.95%	50.43%	64.95%	50.43%
semi-HoETPN (our)	3	3	0.75	**73.87%**	60.57%	72.64%	**59.34%**
semi-HoETPN (our)	2		0.75	73.74%	**61.31%**	**73.24%**	59.28%

**Table 5:** Semi-supervised comparison on the tieredImageNet dataset.

Models	*m*	γ	λ	5way5shot	5way1shot	5way5shot (w/D)	5way1shot (w/D)
PN + Semi [41]				69.37%	50.74%	67.46%	48.67%
Soft k-Means [41]				70.25%	51.52%	68.32%	49.88%
Soft k-Means + Cluster [41]				69.42%	51.85%	67.56%	51.36%
Masked Soft k-Means [41]				69.88%	52.39%	69.08%	51.38%
BR-ProtoNet [36]				79.1%	61.8%	77.4%	60.1%
TPN-semi [25]				71.01%	55.74%	69.93%	53.45%
Semi DSN [32]						70.15%	53.89%
PRWN+Semi [35]				71.06%	59.17%	69.58%	56.59%
semi-HoETPN (our)	3	3	0.75	77.87%	63.87%	77.17%	63.16%
semi-HoETPN (our)	2		0.75	**78.94%**	**65.21%**	**78.45%**	**64.80%**

## Data Availability

The miniImageNet dataset and tieredImageNet dataset can be found at: https://github.com/renmengye/few-shot-ssl-public (accessed on 29 January 2023).
